# Supplementary material for: Assembly mechanism of the inflammasome sensor AIM2 revealed by single molecule analysis
Source: Nat Commun. 2023 Dec 2;14:7957. doi: 10.1038/s41467-023-43691-4 (PMC10693601; doi:10.1038/s41467-023-43691-4)
Supplement: Supplementary file 1 — Supplementary Information [file 41467_2023_43691_MOESM1_ESM.pdf]

SUPPLEMENTARY INFORMATION

Assembly mechanism of the inflammasome sensor AIM2 revealed by single molecule analysis

Meenakshi Sharma and Eva de Alba*

Department of Bioengineering, School of Engineering,
University of California Merced, CA USA

* Corresponding author: Prof. Eva de Alba.
Email: edealbabastarrechea@ucmerced.edu

Contents

1. Supplementary Figures (1 – 10)
2. Supplementary tables (1 – 3)
3. References

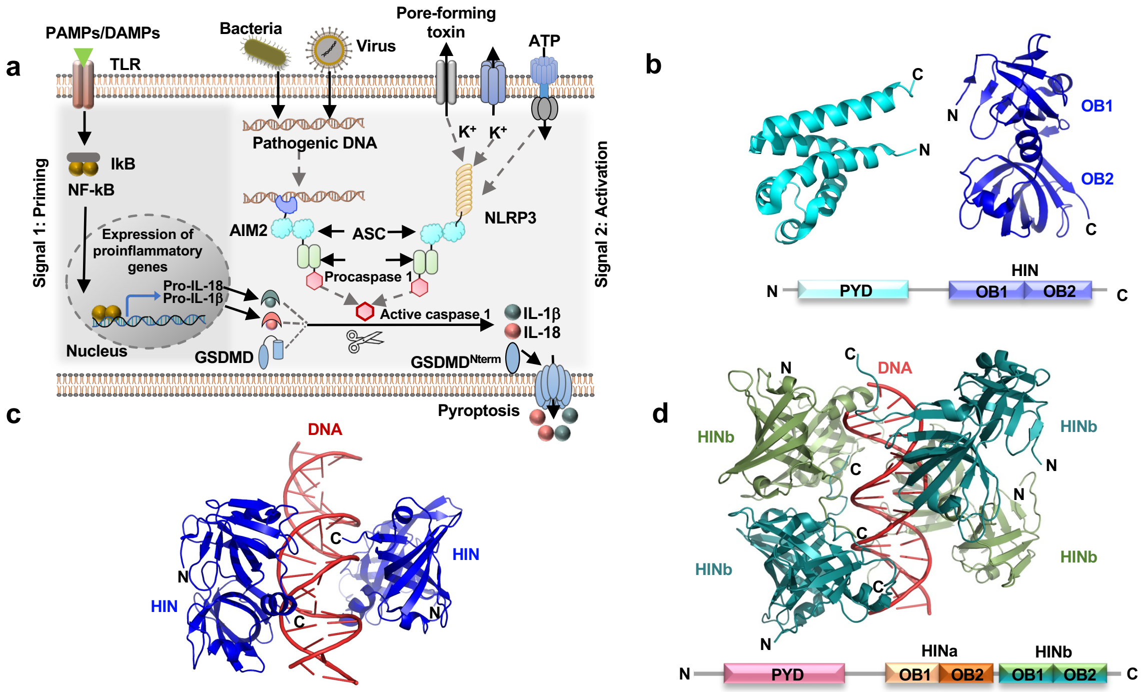

Supplementary Figure 1. Inflammasome operating mode and structure of dsDNA inflammasome sensors. **a** Priming and activation of NLRP3 and AIM2 inflammasomes. The adaptor ASC and maturation products from procaspase 1 activation are indicated. **b** Domain organization and individual three-dimensional structures of AIM2^{HIN} [1] and AIM2^{PYD} [2,3]. **c** Three-dimensional structure of the complex between dsDNA and AIM2^{HIN} [1]. **d** Three-dimensional structure of the complex between dsDNA and the HINb domain of IFI16[1]. IFI16 domain organization (bottom). PYD (Pyrin Domain), HIN (Hematopoietic, Interferon-inducible, Nuclear localization), OB (Oligonucleotide/Oligosaccharide Binding), IL (Interleukin), PAMPs (Pathogen Associated Molecular Patterns), DAMPs (Danger Associated Molecular Patterns), GSDMD (Gasdermin D), GSDMD^{Nterm} (Gasdermin D N-terminal domain), NLRP3 (NLR Family Pyrin Domain Containing 3), ASC (Apoptosis associated Apeck-like protein containing a CARD), AIM2 (Absent In Melanoma 2), NF-κB (Nuclear Factor kappa B), IκB (Inhibitor of nuclear factor kappa B).

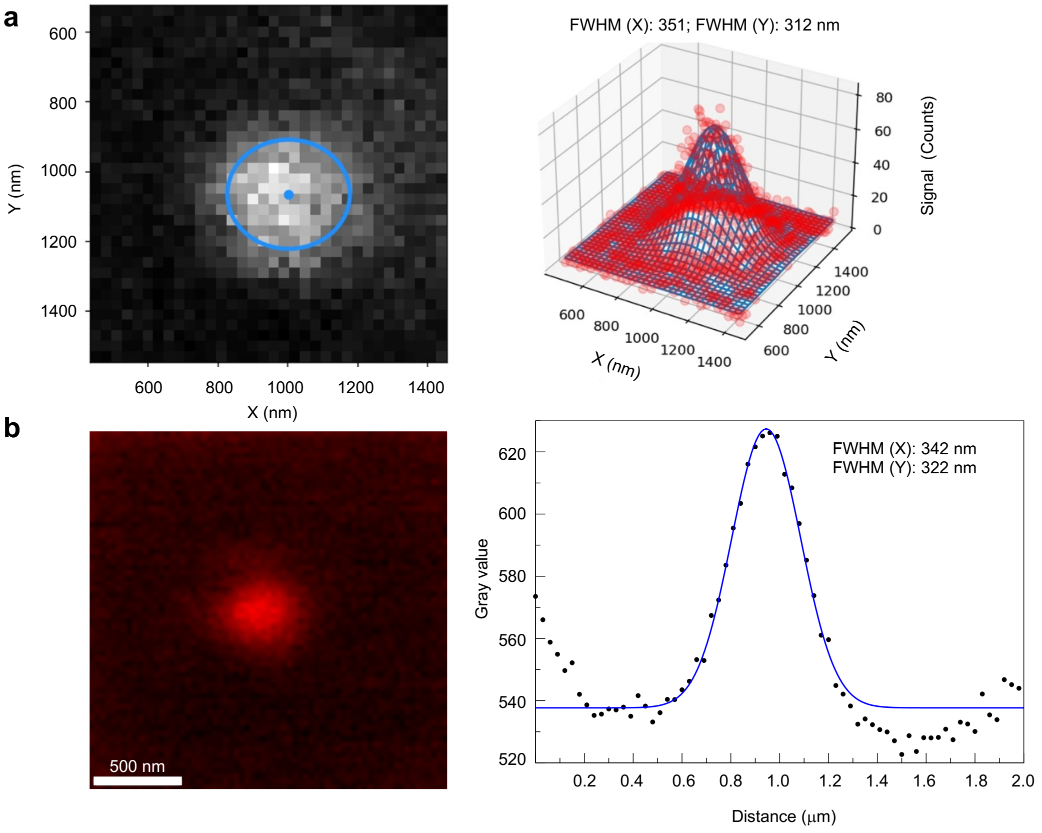

Supplementary Figure 2. Point Spread Function (PSF) analysis of the confocal microscope. a 2D image (left) of a fluorescent bead with a diameter of 100 nm (Excitation at λ = 488 nm) (n = 2) and PSF analysis by LUMICKS (right); **b** 2D image (left) of a 23 nm diameter bead (n = 1). The PSF analysis (Excitation at λ = 638 nm) results in similar full width at half maximum (FWHM) values (inset) for the X- (shown) and Y-axis. Source data are provided as a Source Data file.

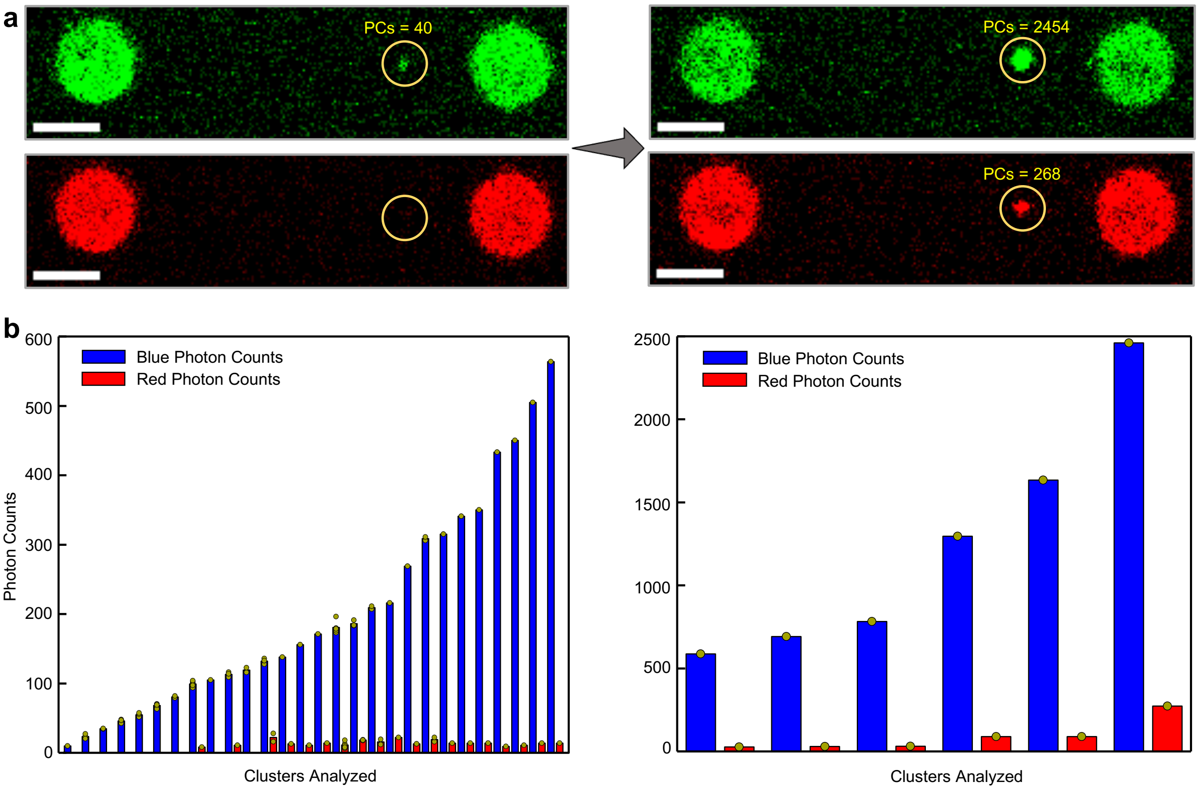

Supplementary Figure 3. Fluorophore oligomerization results in a Stokes shift leading to fluorescence emission at red wavelengths. a Representative 2D confocal fluorescence scans (n = 34) showing emission at 512 nm (green) and 700 nm (red) of clusters emitting different numbers of photon counts (PCs). The arrow indicates that the cluster emitting 40 blue (4 molecules) and 0 red PCs grows to a larger cluster emitting 2454 blue (~ 267 molecules) and 268 red PCs. Scale bars are 3 µm. **b** Plots representing the difference between blue and red PCs (number of clusters analyzed, n = 75). Oligomers emitting less than 100 blue PCs do not show emission in the red (left plot). Clusters emitting less than 600 blue PCs show an average emission of 14 ± 4 red photons (left plot). Oligomers emitting more than 600 PCs do not show a linear increase in red emission as blue emission increases (right plot). Green circles represent individual measurements. Source data are provided as a Source Data file.

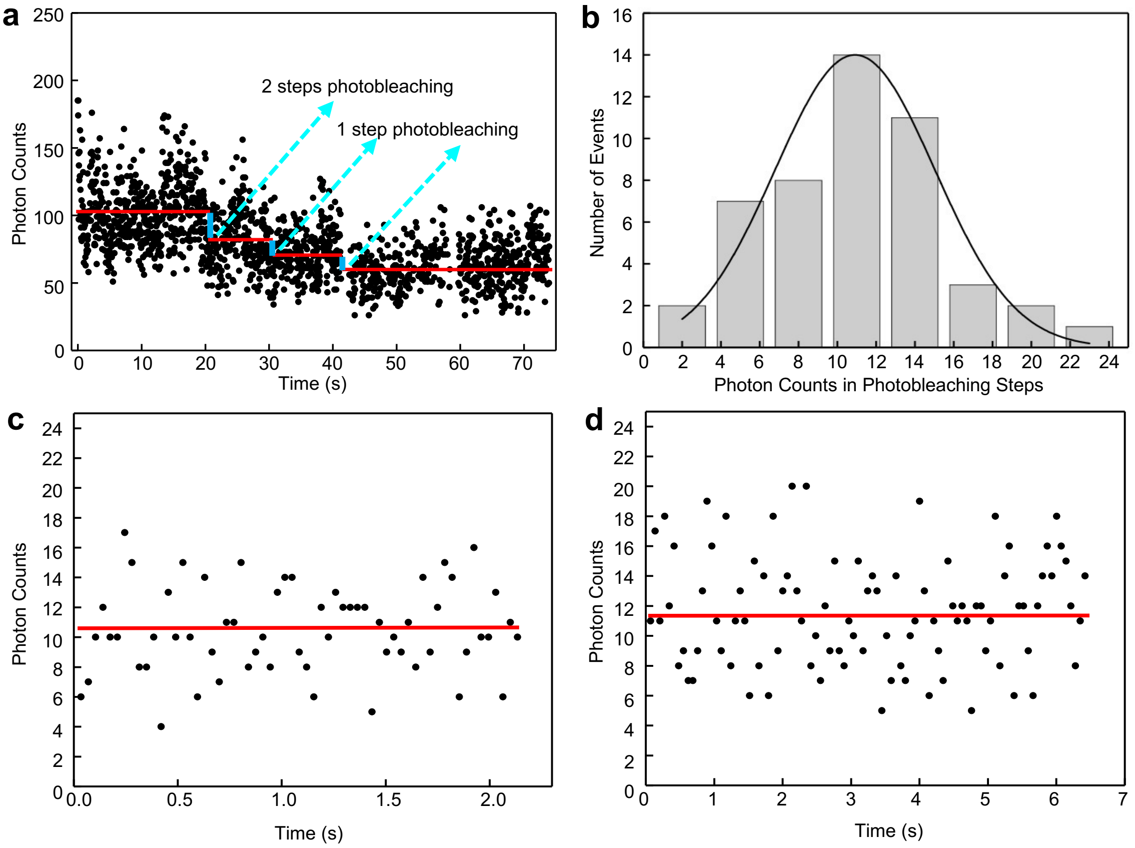

Supplementary Figure 4. Photobleaching leads to a decrease with time of fluorescence intensity emitted by AIM2 oligomers. a Example of confocal fluorescence intensity decreasing with time (traces; n = 22) due to photobleaching of a single trace extracted from a kymograph. Red lines represent average values of the intensity. The step to the left corresponds to two-fluorophore photobleaching (cyan). The second and third steps correspond to single fluorophore photobleaching (cyan). This analysis is done by visual inspection. **b** Distribution of single step photobleaching events (n = 48) extracted from kymographs. Fitting to a Gaussian function results in an average number of 11 ± 4 photon counts for a single fluorophore. The goodness of the fit is represented by an R-square of 0.95 and RMSE (root mean squared error) of 0.42. **c, d** Examples of two traces (n = 314) corresponding to single fluorophores with an average of approximately 11 photon counts (indicated by horizontal red lines) in agreement with the value determined from the distribution in **b**. Source data are provided as a Source Data file.

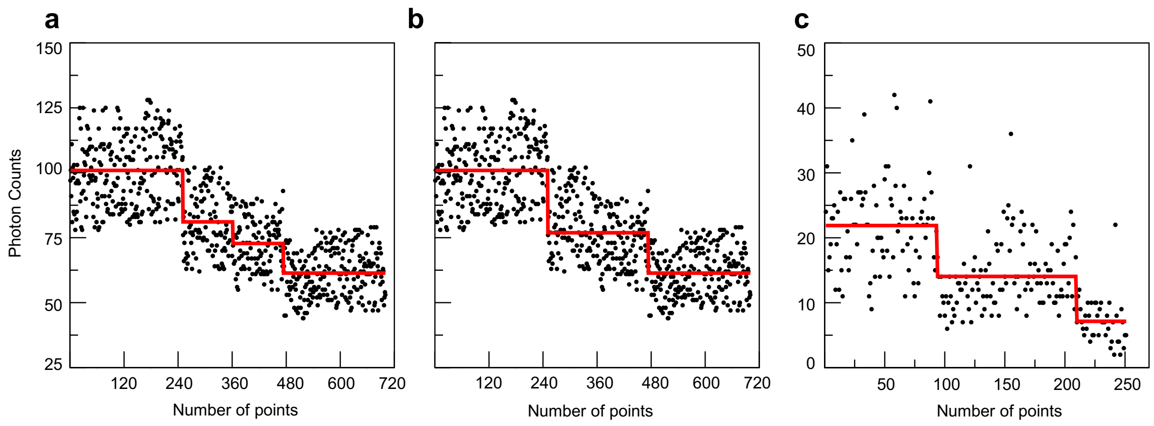

Supplementary Figure 5. Automated detection method of photobleaching steps. a, b Trajectory shown in Supplementary Figure 4a after filtering data outside the range of average photon counts (PCs) ± σ (standard deviation) calculated in intervals of 10 seconds. Photobleaching steps detected by Autostepfinder[4] in manual (**a**) and automatic (**b**) modes result in identical quality of fit. **c** A representative trajectory (raw data, n = 22) leads to two steps using Autostepfinder. Automatically detected steps in **a** are 19.8, 8.3, 11.4 PCs; **b**, 23.98, 15.5 PCs; and **c**, 7.8, 6.9 PCs. The original steps found by visual inspection of these trajectories are **a**, 20.4, 10, 10.5 PCs; and **c**, 8.8, 6.1 PCs. Source data are provided as a Source Data file.

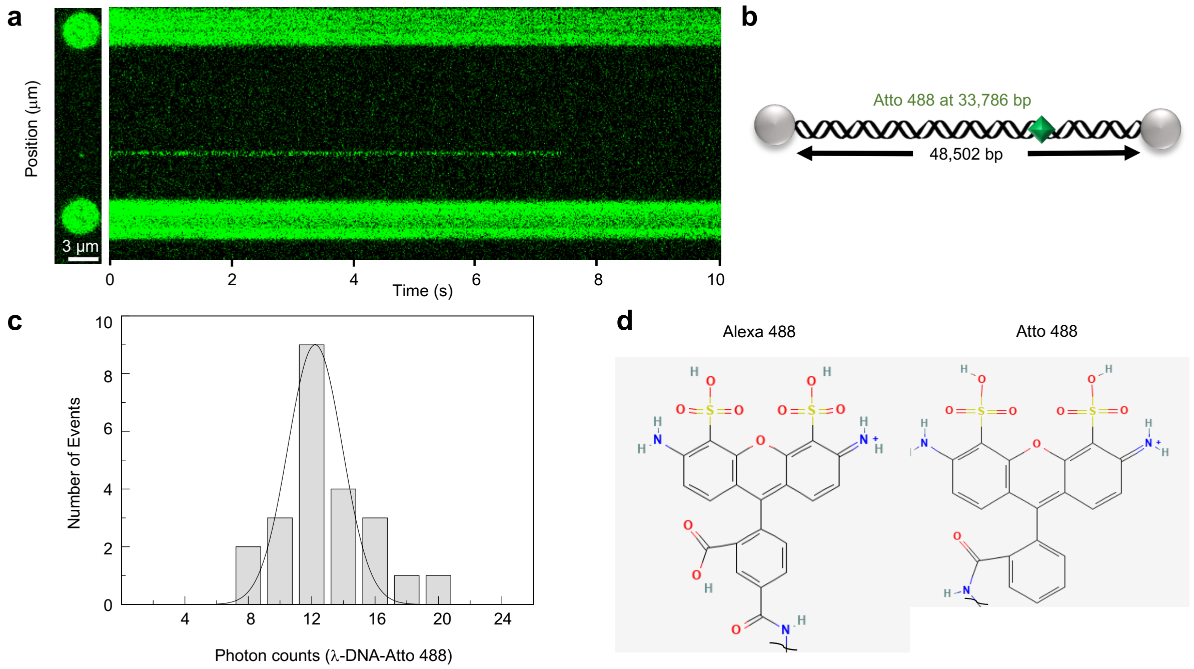

Supplementary Figure 6. Photon counts emitted by a single fluorophore. a 2D image of single Atto 488 attached to λ-DNA (n = 27) and corresponding kymograph (n = 23; excitation at 488 nm). **b** Schematic representation of single fluorophore Atto 488 attached to base pair 33,786 in λ-DNA. **c** Photon count analysis of traces (n = 23) such as the one shown in **a**. The fitting to a Gaussian distribution indicates that a single fluorophore emits 12 ± 2 photon counts ($\bar{x} \pm \sigma$). **d** Molecular structures of Alexa 488[5] and Atto 488[6] show great similarity. Source data are provided as a Source Data file.

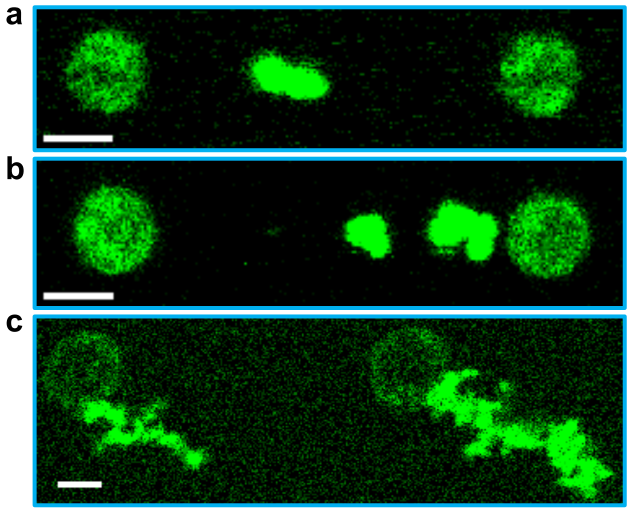

Supplementary Figure 7. Large AIM2 self-assemblies in the presence and absence of dsDNA. a, b Representative 2D confocal fluorescence scans showing large AIM2 oligomers bound to the dsDNA (n = 17) (bead diameter and scale bars are 3 µm). **c** 2D confocal fluorescence scan in the absence of dsDNA (n = 1) showing large AIM2 oligomers trapped with beads (bead diameter is ~ 4 µm and scale bar is 3 µm).

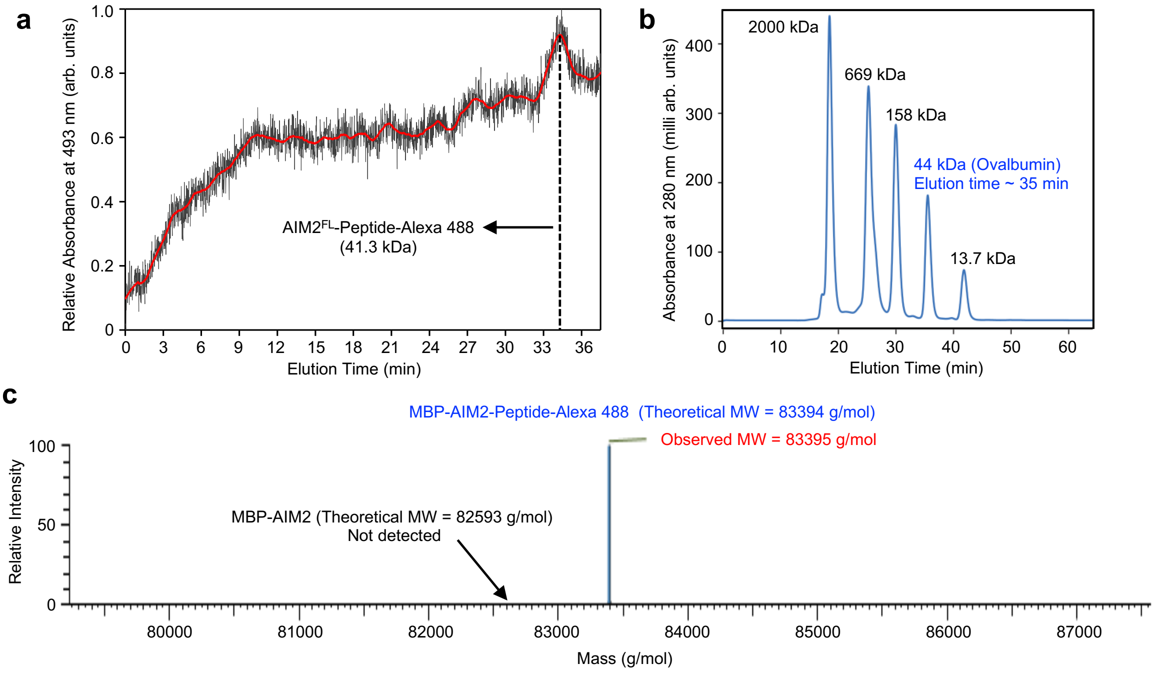

Supplementary Figure 8. AIM2 is monomeric during purification by size exclusion chromatography. a Size exclusion chromatogram is a representative of multiple purification experiments ($n > 8$) showing the elution time and absorbance at 493 nm of full-length AIM2 labeled with peptide (GGGC) carrying Alexa 488 (Matrix: Superdex 200 increase 10/300 GL, GE Healthcare) after cleavage of 450 nM MBP-AIM2-Peptide-Alexa 488 with TEV protease. The detector signal was multiplied by a factor of 500. **b** Chromatogram reported by GE Healthcare for a mixture of protein standards including ovalbumin with a molecular mass close to AIM2-peptide-Alexa 488. Ovalbumin's elution time is very close to that of AIM2-peptide-Alexa 488. **c** Mass spectrometry data of the labeling of MBP-AIM2 with Peptide-Alexa 488 by sortase transpeptidation. The observed and theoretical masses match within 1 Da (in red and blue, respectively). The black arrow points to where a signal corresponding to the mass of the unlabeled protein should be expected. The unlabeled protein was not detected. This result indicates that the labeling reaction reaches approximately 100% efficiency. Source data are provided as a Source Data file.

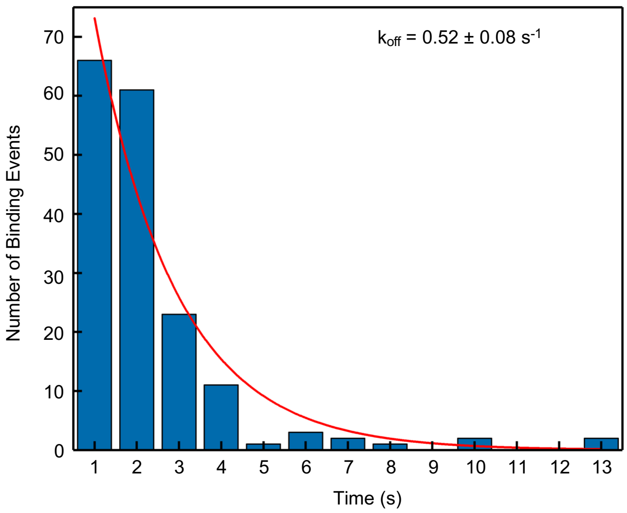

Supplementary Figure 9. DNA stretching leads to an increase in the AIM2-dsDNA dissociation rate constant. Dwell time analysis of single AIM2 molecule traces (n = 172) in kymographs acquired at 40 pN (λ-DNA stretched to 16.5 µm, 10 nM protein concentration). The red line represents the fittings to a single exponential function reporting the dissociation rate constant (k_{off}). The goodness of fit is represented by R-square and RMSE (root mean squared error) values of 0.93 and 5.99. Source data are provided as a Source Data file.

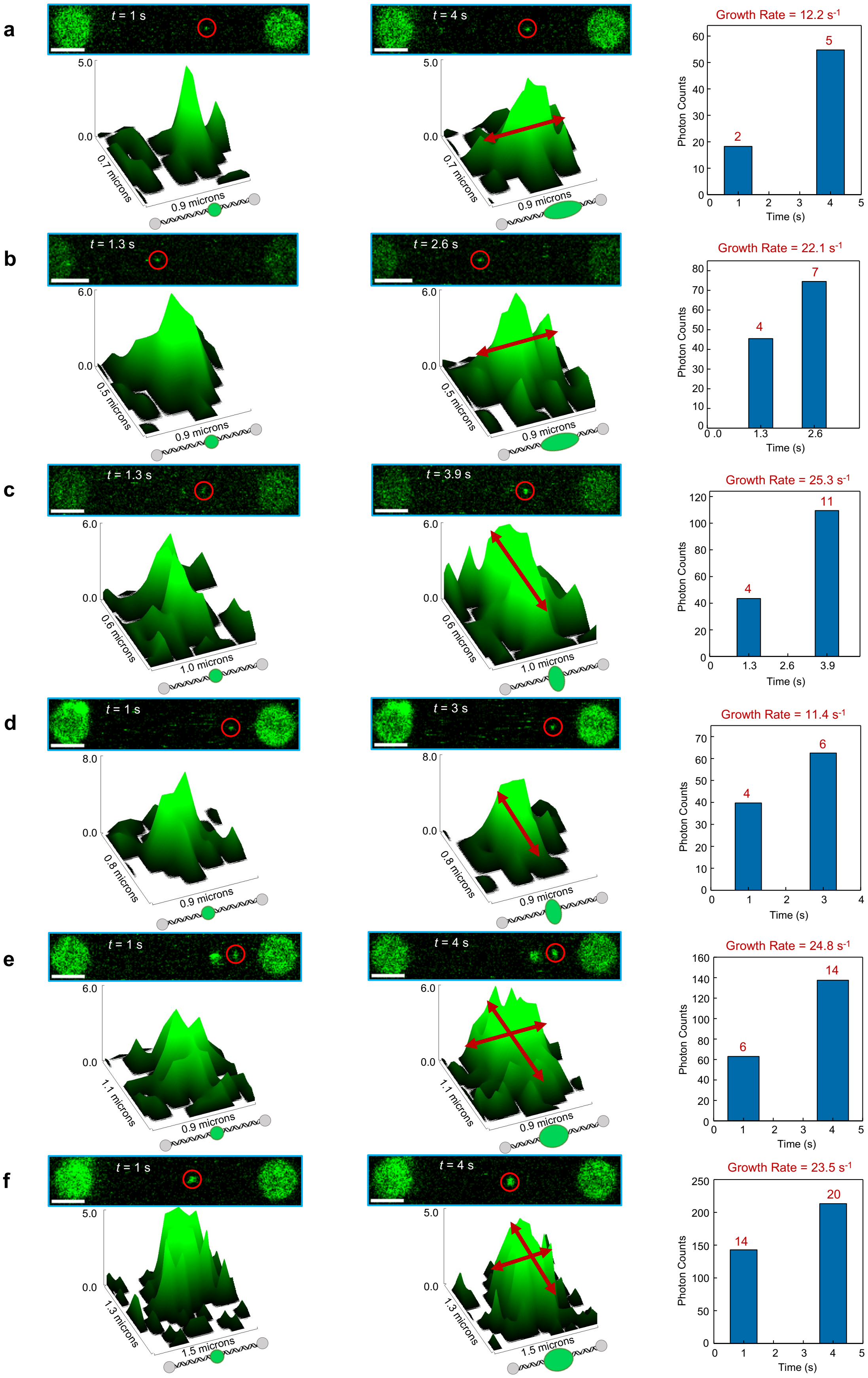
a
t = 1 s
t = 4 s
5.0
0.0
0.7 microns
0.9 microns
Growth Rate = 12.2 s-1
Photon Counts
Time (s)
2
5
b
t = 1.3 s
t = 2.6 s
6.0
0.0
0.5 microns
0.9 microns
Growth Rate = 22.1 s-1
4
7
c
t = 1.3 s
t = 3.9 s
6.0
0.0
0.6 microns
1.0 microns
Growth Rate = 25.3 s-1
4
11
d
t = 1 s
t = 3 s
8.0
0.0
0.8 microns
0.9 microns
Growth Rate = 11.4 s-1
4
6
e
t = 1 s
t = 4 s
6.0
0.0
1.1 microns
0.9 microns
Growth Rate = 24.8 s-1
6
14
f
t = 1 s
t = 4 s
5.0
0.0
1.3 microns
1.5 microns
Growth Rate = 23.5 s-1
14
20

Supplementary Figure 10. Direction of growth and growth rate of AIM2 oligomers bound to dsDNA. Left and middle: 2D confocal frames of movies ($n = 45$) acquired at two time points (white) showing AIM2 oligomers (red circles) bound to dsDNA. The corresponding surface plots illustrate the direction of oligomer growth (red arrows). Grey circles represent the trapped beads tethered by dsDNA (black chain) and green circles represent AIM2 oligomers. Right: Fluorescence intensity increase (blue bars) and the corresponding number of starting and final number of molecules (red numbers) of the clusters at different times. The cluster growth rate (red) is indicated on top of each plot. AIM2 concentration values are, 13 nM (**a, e, f**), 10 nM (**b, c**) 2 nM (**d**). All scale bars are 3 μm. Source data are provided as a Source Data file.

Supplementary Table 1: Residence times of AIM2 oligomers with different numbers of protomers on a single dsDNA molecule

Cluster size (Number of protomers)	Residence time (s)*
1	3.3
2	2.9
3	> 680
4	> 680
5	> 751
6	> 627
7	> 1516
8	> 680
10	> 627
11	> 619
14	> 619
17	> 951
64	> 1105
113	> 751
205	> 951
231	> 668
234	>1516
309	> 951
333	> 668
335	> 643
339	> 643
361	> 668
370	> 951
514	> 643

* “>” indicates that the oligomer remains attached to the single dsDNA for the total length of the kymograph.

Supplementary Table 2: Unbound times (t_{on}) of single AIM2 molecules on dsDNA obtained from kymographs acquired at 1 nM protein concentration and 600 s total observation time

Kymograph	Number of traces analyzed	Unbound time, t_{on} (s)	Average t_{on} (s)
1	26	443.2	551.0
2	21	483.6	
3	8	580.7	
4	8	519.2	
5	7	527.9	
6	5	574.8	
7	5	528.3	
8	5	571.6	
9	4	587.4	
10	3	584.7	
11	3	589.0	
12	3	585.4	
13	2	587.7	

Supplementary Table 3: Unbound times (t_{on}) of single AIM2 molecules on dsDNA obtained from kymographs acquired at 5 nM protein concentration and 600 s total observation time

Kymograph	Number of traces analyzed	Unbound time, t_{on} (s)	Average t_{on} (s)
1	14	527.6	544.3
2	14	563.6	
3	13	546.6	
4	13	528.5	
5	11	576.2	
6	11	546.5	
7	9	524.5	
8	9	530.4	
9	8	527.1	
10	4	581.6	
11	3	534.9	

References

1. Jin, T. *et al.* Structures of the HIN Domain:DNA Complexes Reveal Ligand Binding and Activation Mechanisms of the AIM2 Inflammasome and IFI16 Receptor. *Immunity* **36**, 561–571 (2012).
2. Jin, T., Perry, A., Smith, P., Jiang, J. & Xiao, T. S. Structure of the Absent in Melanoma 2 (AIM2) Pyrin Domain Provides Insights into the Mechanisms of AIM2 Autoinhibition and Inflammasome Assembly. *Journal of Biological Chemistry* **288**, 13225–13235 (2013).
3. Lu, A., Kabaleeswaran, V., Fu, T., Magupalli, V. G. & Wu, H. Crystal Structure of the F27G AIM2 PYD Mutant and Similarities of Its Self-Association to DED/DED Interactions. *J Mol Biol* **426**, 1420–1427 (2014).
4. Loeff, L., Kerssemakers, J. W. J., Joo, C. & Dekker, C. AutoStepfinder: A fast and automated step detection method for single-molecule analysis. *Patterns* **2**, 100256 (2021).
5. https://pubchem.ncbi.nlm.nih.gov/compound/Alexa-488
6. https://pubchem.ncbi.nlm.nih.gov/compound/102227067
