# Supplementary material for: Assembly mechanism of the inflammasome sensor AIM2 revealed by single molecule analysis
Source: Nat Commun. 2023 Dec 2;14:7957. doi: 10.1038/s41467-023-43691-4 (PMC10693601; doi:10.1038/s41467-023-43691-4)
Supplement: Supplementary file 3 — Description of Additional Supplementary Files [file 41467_2023_43691_MOESM3_ESM.pdf]

Description of Additional Supplementary Files

File Name: Supplementary Data 1
Description: Table listing company names and catalog numbers of commercial reagents used in the study.

File Name: Supplementary Movie 1
Description: **Diffusion of MBP-AIM2 oligomers along the dsDNA.** Representative movie (*n* = 5) showing the diffusion of MBP-AIM2 oligomers (250 pM) on dsDNA at 50 mM NaCl.
