# Supplementary material for: Assembly mechanism of the inflammasome sensor AIM2 revealed by single molecule analysis
Source: Nat Commun. 2023 Dec 2;14:7957. doi: 10.1038/s41467-023-43691-4 (PMC10693601; doi:10.1038/s41467-023-43691-4)
Supplement: Supplementary file 6 — Reporting Summary [file 41467_2023_43691_MOESM6_ESM.pdf]

nature portfolio

Corresponding author(s): Eva de Alba
Last updated by author(s): Oct 19, 2023

Reporting Summary

Nature Portfolio wishes to improve the reproducibility of the work that we publish. This form provides structure for consistency and transparency in reporting. For further information on Nature Portfolio policies, see our Editorial Policies and the Editorial Policy Checklist.

Statistics

For all statistical analyses, confirm that the following items are present in the figure legend, table legend, main text, or Methods section.

n/a	Confirmed	
☐	☒	The exact sample size (n) for each experimental group/condition, given as a discrete number and unit of measurement
☐	☒	A statement on whether measurements were taken from distinct samples or whether the same sample was measured repeatedly
☒	☐	The statistical test(s) used AND whether they are one- or two-sided<br>*Only common tests should be described solely by name; describe more complex techniques in the Methods section.*
☒	☐	A description of all covariates tested
☒	☐	A description of any assumptions or corrections, such as tests of normality and adjustment for multiple comparisons
☐	☒	A full description of the statistical parameters including central tendency (e.g. means) or other basic estimates (e.g. regression coefficient) AND variation (e.g. standard deviation) or associated estimates of uncertainty (e.g. confidence intervals)
☒	☐	For null hypothesis testing, the test statistic (e.g. F, t, r) with confidence intervals, effect sizes, degrees of freedom and P value noted<br>*Give P values as exact values whenever suitable.*
☒	☐	For Bayesian analysis, information on the choice of priors and Markov chain Monte Carlo settings
☒	☐	For hierarchical and complex designs, identification of the appropriate level for tests and full reporting of outcomes
☒	☐	Estimates of effect sizes (e.g. Cohen's d, Pearson's r), indicating how they were calculated

Our web collection on statistics for biologists contains articles on many of the points above.

Software and code

Policy information about availability of computer code

Data collection	Data acquisition was performed using BlueLake software version 2.3 (LUMICKS)
Data analysis	Confocal images and kymographs were processed and analyzed with the Pylake software (https://lumicks-pylake.readthedocs.io/en/stable) version 0.13.2 and with scripts retrieved from the Harbor platform (https://harbor.lumicks.com).<br>Intensity and area in confocal images were further analyzed with Fiji (https://imagej.net/software/fiji/downloads) version 1.0 or ImageJ2 version 2.9.0.<br>Graphs were plotted and analyzed using OriginLab (https://www.originlab.com) version 2017 and qtGrace (https://sourceforge.net/projects/qtgrace) version 0.2.6.

For manuscripts utilizing custom algorithms or software that are central to the research but not yet described in published literature, software must be made available to editors and reviewers. We strongly encourage code deposition in a community repository (e.g. GitHub). See the Nature Portfolio guidelines for submitting code & software for further information.

Data

Policy information about availability of data

All manuscripts must include a data availability statement. This statement should provide the following information, where applicable:

- Accession codes, unique identifiers, or web links for publicly available datasets
- A description of any restrictions on data availability
- For clinical datasets or third party data, please ensure that the statement adheres to our policy

All data are available within this manuscript and its supplementary information files. Original files in "HDF5" format corresponding to 2D confocal scans and kymographs are available from the corresponding author upon request due to the large file sizes. Source data are provided with this paper.

Research involving human participants, their data, or biological material

Policy information about studies with human participants or human data. See also policy information about sex, gender (identity/presentation), and sexual orientation and race, ethnicity and racism.

Reporting on sex and gender	N/A
Reporting on race, ethnicity, or other socially relevant groupings	N/A
Population characteristics	N/A
Recruitment	N/A
Ethics oversight	N/A

Note that full information on the approval of the study protocol must also be provided in the manuscript.

Field-specific reporting

Please select the one below that is the best fit for your research. If you are not sure, read the appropriate sections before making your selection.

[x] Life sciences   [ ] Behavioural & social sciences   [ ] Ecological, evolutionary & environmental sciences

For a reference copy of the document with all sections, see nature.com/documents/nr-reporting-summary-flat.pdf

Life sciences study design

All studies must disclose on these points even when the disclosure is negative.

Sample size	Sample size was determined based on similar studies in this field (references 41, 42, 47, 48, 49, 52).
Data exclusions	Data were excluded for the first bin in figures 3d and e due to the observed smaller values indicating the data are outliers.
Replication	Representative data are shown for each experiment. Each experiment is reproducible.<br>Figure 1b; n>278<br>Figure 1c; n=106<br>Figure 2a; n=155<br>Figure 3d; n=314<br>Figure 3e; n=100<br>Figure 3f; n=109<br>Figure 4a-c; n=45<br>Figure 6a-c; n=45<br>Figure 7c; n=70<br>Figure 7d; n=40<br>Suppl. Figure 2a; n=2<br>Suppl. Figure 2b; n=1<br>Suppl. Figure 3a; n=34<br>Suppl. Figure 3b; n=75<br>Suppl. Figure 4a; n=22<br>Suppl. Figure 4b; n=48<br>Suppl. Figure 4c, d; n=314<br>Suppl. Figure 6a; n=27 for 2D scans, n=23 for kymographs<br>Suppl. Figure 6b; n=23<br>Suppl. Figure 7a, b; n=17<br>Suppl. Figure 7c; n=1

Suppl. Figure 8a; n>8
Suppl. Figure 9; n=172
Suppl. Figure 10; n=45
Suppl. video 1; n=5

Randomization: Randomization was not required to avoid bias during data analysis.

Blinding: The authors are not blinded to the group allocation and analysis.

Reporting for specific materials, systems and methods

We require information from authors about some types of materials, experimental systems and methods used in many studies. Here, indicate whether each material, system or method listed is relevant to your study. If you are not sure if a list item applies to your research, read the appropriate section before selecting a response.

Materials & experimental systems

n/a	Involved in the study
☒	☐ Antibodies
☒	☐ Eukaryotic cell lines
☒	☐ Palaeontology and archaeology
☒	☐ Animals and other organisms
☒	☐ Clinical data
☒	☐ Dual use research of concern
☒	☐ Plants

Methods

n/a	Involved in the study
☒	☐ ChIP-seq
☒	☐ Flow cytometry
☒	☐ MRI-based neuroimaging
